# 1,2-Bis[5-(4-cyano­phen­yl)-2-methyl-3-thien­yl]-3,3,4,4,5,5-hexa­fluoro­cyclo­pent-1-ene: a photochromic diaryl­ethene compound

**DOI:** 10.1107/S1600536808011653

**Published:** 2008-04-30

**Authors:** Gang Liu, Qidong Tu, Qing Zhang, Congbin Fan, Tianshe Yang

**Affiliations:** a Jiangxi Key Laboratory of Organic Chemistry, Jiangxi Science & Technology Normal University, Nanchang 330013, People’s Republic of China

## Abstract

The mol­ecules of the title compound, C_29_H_16_F_6_N_2_S_2_, a photochromic dithienylethene with 4-cyano­phenyl substituents, adopt an anti­parallel arrangement that is reponsible for photoactivity. The mol­ecule lies on a twofold rotation axis. The dihedral angle between the nearly planar cyclo­pentenyl and heteroaryl rings is 142.5 (3)°, and that between the heteroaryl and benzene rings is 22.4 (3)°. The distance between the heteroaryl rings of adjacent mol­ecules is 3.601 (2) Å, indicating a π–π interaction.

## Related literature

For a review of dithienylethyl­enes as photochromic compounds, see: Irie (2000 ▶). For phenyl-substituted derivatives, see: Pu, Liu *et al.* (2005 ▶); Pu, Yang *et al.* (2005 ▶). For another similar structure, see: Kobatake *et al.* (2004 ▶). For the manifestation of possible photochromic activity in relation to the conformation, see: Woodward & Hoffmann (1970 ▶).
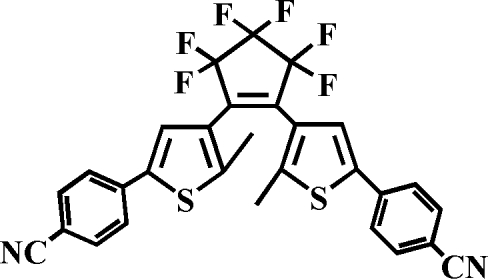


## Experimental

### 

#### Crystal data

C_29_H_16_F_6_N_2_S_2_
*M* *_r_* = 570.56
Monoclinic, 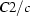

*a* = 24.987 (10) Å
*b* = 9.276 (4) Å
*c* = 10.774 (4) Å
β = 95.911 (7)°
*V* = 2483.8 (17) Å^3^
*Z* = 4
Mo *K*α radiation
μ = 0.28 mm^−1^
*T* = 113 (2) K
0.36 × 0.20 × 0.10 mm

#### Data collection

Rigaku Saturn diffractometer
Absorption correction: multi-scan (Jacobson, 1998 ▶) *T*_min_ = 0.905, *T*_max_ = 0.972
9917 measured reflections
2437 independent reflections
2130 reflections with *I* > 2σ(*I*)
*R*_int_ = 0.032

#### Refinement

*R*[*F* ^2^ > 2σ(*F* ^2^)] = 0.057
*wR*(*F* ^2^) = 0.145
*S* = 1.06
2437 reflections
180 parameters
H-atom parameters constrained
Δρ_max_ = 0.90 e Å^−3^
Δρ_min_ = −0.65 e Å^−3^

### 

Data collection: *CrystalClear* (Rigaku/MSC, 2001 ▶); cell refinement: *CrystalClear*; data reduction: *CrystalStructure* (Rigaku/MSC, 2004 ▶); program(s) used to solve structure: *SHELXS97* (Sheldrick, 2008 ▶); program(s) used to refine structure: *SHELXL97* (Sheldrick, 2008 ▶); molecular graphics: *SHELXTL* (Sheldrick, 2008 ▶); software used to prepare material for publication: *CrystalStructure*.

## Supplementary Material

Crystal structure: contains datablocks I, global. DOI: 10.1107/S1600536808011653/ng2448sup1.cif

Structure factors: contains datablocks I. DOI: 10.1107/S1600536808011653/ng2448Isup2.hkl

Additional supplementary materials:  crystallographic information; 3D view; checkCIF report

## Figures and Tables

**Fig. 1.**
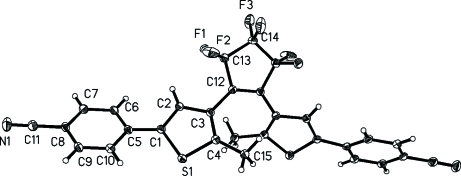
The molecular structure of (Ia) with 35% probability ellipsoids, showing the atomic numbering scheme.

**Fig. 2.**
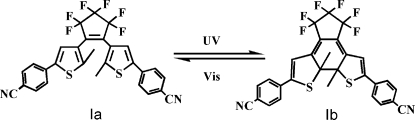
Interconversion of compound (Ia) and compound (Ib).

**Fig. 3.**
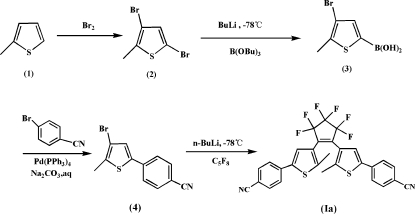
Synthetical method of compound (Ia).

## supplementary crystallographic information

### Comment

Dithienylethenes are the most promising organic photochromic compounds for photoelectronic devices(Irie, 2000).Dithienylethenes bearing terminal phenyl groups are of special interest, because phenyl group can be substituted by many electron-donating or electron-withdrawing groups that influence the properties(Pu, Yang,Xu *et al.*,2005). In order to investigate the substituent effect at the *para*-position on the photochemical properties, we have now synthesized the title compound, (Ia), and its structure is presented in this paper.

To the best of our knowledge,this is the first symmetrical dithienylethene compound with phenyl groups bearing *para* substituent. The molecular structure of (Ia) is shown in Fig. 1 and selected geometric parameters are given in Table 1.

The molecule contains two thiophene rings substituted by two *para*-cyanophenyl rings in a photoactive antiparallel conformation. In the cyclopent-1-ene ring, the C12=C12A bond is clearly a double bond, and the other bonds in the ring are clearly single bonds(see Table 1). The two thiophene rings are linked by the C12=C12A double bond.The two methyl groups are located on different sides of the double bond and are thus *trans* with respect to the double bond. Such a configuration is crucial for the compound to exhibit photochromic and photoinduced properties (Woodward & Hoffmann, 1970). The dihedral angles between the least-squares plane of the atoms of the central cyclopent-1-ene ring and the adjacent thiophene rings are 142.5 (3)°, and those between thiophene rings and the adjacent benzene rings are both 22.4 (3)°.The distance between the two reactive C atoms (C4_C4A) is 3.601 (2) Å. This distance indicates that the crystal can undergo photochromism in the crystalline phase because the photochromic reactivity of crystals depends on the distance between the reactive C atoms being less than 4.2 Å(Kobatake *et al.*, 2004).

Upon irradiation with 313 nm light, colorless single crystals of (Ia) turned to blue rapidly, and the blue color remained stable in the dark. When the blue crystals were dissolved in hexane, the solution also remained blue.The absorption maximum of this solution is observed at a wavelength of 596 nm, consistent with the presence of the closed-ring isomer, (Ib).This result suggests that the title compound undergoes a photochromic reaction to produce the closed-ring molecule of (Ib) in the single-crystal phase. We have not, so far, been able to determine the crystal structure of (Ib). Furthermore, upon irradiation with wavelengths longer than 450 nm, the blue crystal changes back to colorless, and the absorption spectrum of a hexane solution of the colorless crystals is the same as that of a solution of the open-ring form, (Ia), with the absorption maximum at 314 nm.

### Experimental

The title compound was originally derived from 2-methylthiophene(1). 3-Bromo-2-methyl-5-(4-cyanophenyl)thiophene, (4) (4.60 g, 16.5 mmol), in 78.8% yield was synthesized by reacting(3) (4.62 g, 20.9 mmol) (Pu, Liu *et al.*, 2005) with 4-cyano-brombenzene (3.82 g, 21.0 mmol) in the presence of Pd(PPh_3_)_4_ (0.5 g) and Na_2_CO_3_ (2 mol/*L*, 30 ml) in tetrahydrofuran (THF, 80 ml) for 16 h at 343 K. To a stirred THF solution (60 ml) of (4) (2.21 g, 7.96 mmol) 3.3 ml of n-BuLi/hexane solution (2.5 *M*, 8.2 mmol) was slowly added at 195 K under a nitrogen atmosphere. 30 min later, octa-fluorocyclopentene (0.54 ml, 4.0 mmol) was added and the mixture was stirred for 2 h. The reaction mixture was extracted with diethyl ether and evaporated *in vacuo*, then purified by column chromatography (petroleum ether) to give the title compound (1.21 g, 2.12 mmol) in 53.3% yield. The compound crystallized from hexane at room temperature and produced single crystals suitable for X-ray analysis. The structure of (Ia) was confirmed by melting point, IR and NMR. Analysis calculated for C_29_H_16_F_6_N_2_S_2_, m.p.: 485.7 k ^1^H NMR (400 MHz, CDCl_3_) δ 2.03 (s, 6H),δ 7.41 (s, 2H), δ 7.64, 7.70 (d, 8H, J = 8.0 Hz); ^13^C NMR (400 MHz, CDCl_3_) δ 14.67, 111.33, 118.47, 124.41, 125.87, 126.28, 132.86, 137.34, 140.20, 143.42; IR (n, KBr, cm-1): 743, 831, 846, 887, 987, 1054, 1108, 1184, 1267, 1309, 1338, 1385, 1413, 1438, 1438, 1508, 1551, 1603, 1633, 2223.

### Refinement

H atoms were positioned theoretically and allowed to ride on their parent atoms in the final refinement[C—H = 0.93–0.96Å and *U*_iso_(H) = 1.2Ueq(C) or 1.5Ueq(methyl C)].The methyl groups were treated as rigid groups and allowed to rotate about the C—C bond.

### Crystal data

**Table:**

C_29_H_16_F_6_N_2_S_2_	*F*_000_ = 1160
*M**_r_* = 570.56	*D*_x_ = 1.526 Mg m^−^^3^
Monoclinic, *C*2/*c*	Mo *K*α radiation λ = 0.71070 Å
Hall symbol: -C2yc	Cell parameters from 3563 reflections
*a* = 24.987 (10) Å	θ = 1.6–27.9º
*b* = 9.276 (4) Å	µ = 0.28 mm^−^^1^
*c* = 10.774 (4) Å	*T* = 113 (2) K
β = 95.911 (7)º	Block, colorless
*V* = 2483.8 (17) Å^3^	0.36 × 0.20 × 0.10 mm
*Z* = 4	

### Data collection

**Table:**

Rigaku Saturn diffractometer	2437 independent reflections
Radiation source: rotating anode	2130 reflections with *I* > 2σ(*I*)
Monochromator: confocal	*R*_int_ = 0.032
Detector resolution: 7.31 pixels mm^-1^	θ_max_ = 26.0º
*T* = 113(2) K	θ_min_ = 1.6º
ω scans	*h* = −30→30
Absorption correction: multi-scan(Jacobson, 1998)	*k* = −11→11
*T*_min_ = 0.905, *T*_max_ = 0.972	*l* = −12→13
9917 measured reflections	

### Refinement

**Table:**

Refinement on *F*^2^	Hydrogen site location: inferred from neighbouring sites
Least-squares matrix: full	H-atom parameters constrained
*R*[*F*^2^ > 2σ(*F*^2^)] = 0.057	*w* = 1/[σ^2^(*F*_o_^2^) + (0.0692*P*)^2^ + 6.3864*P*] where *P* = (*F*_o_^2^ + 2*F*_c_^2^)/3
*wR*(*F*^2^) = 0.145	(Δ/σ)_max_ = 0.001
*S* = 1.07	Δρ_max_ = 0.90 e Å^−^^3^
2437 reflections	Δρ_min_ = −0.65 e Å^−^^3^
180 parameters	Extinction correction: SHELXL97 (Sheldrick, 2008), Fc^*^=kFc[1+0.001xFc^2^λ^3^/sin(2θ)]^-1/4^
Primary atom site location: structure-invariant direct methods	Extinction coefficient: 0.0031 (9)
Secondary atom site location: difference Fourier map	

### Special details

**Table:**

Refinement. Refinement of *F*^2^ against ALL reflections. The weighted *R*-factor *wR* and goodness of fit *S* are based on *F*^2^, conventional *R*-factors *R* are based on *F*, with *F* set to zero for negative *F*^2^. The threshold expression of *F*^2^ > σ(*F*^2^) is used only for calculating *R*-factors(gt) *etc*. and is not relevant to the choice of reflections for refinement. *R*-factors based on *F*^2^ are statistically about twice as large as those based on *F*, and *R*- factors based on ALL data will be even larger.

### Fractional atomic coordinates and isotropic or equivalent isotropic displacement parameters (Å^2^)

**Table:**

	*x*	*y*	*z*	*U*_iso_*/*U*_eq_	
S1	0.12440 (3)	0.06080 (7)	0.81522 (6)	0.0293 (2)	
F1	0.04313 (8)	0.57014 (18)	0.94258 (16)	0.0469 (5)	
F2	0.09200 (7)	0.57943 (19)	0.7907 (2)	0.0607 (7)	
F3	−0.02249 (10)	0.7331 (3)	0.8302 (2)	0.0846 (10)	
N1	0.34207 (11)	0.1164 (3)	1.4057 (3)	0.0492 (7)	
C1	0.13504 (10)	0.1761 (3)	0.9418 (2)	0.0252 (6)	
C2	0.09893 (10)	0.2871 (3)	0.9294 (2)	0.0248 (6)	
H2	0.0981	0.3621	0.9893	0.030*	
C3	0.06264 (10)	0.2802 (3)	0.8183 (2)	0.0244 (6)	
C4	0.07183 (10)	0.1622 (3)	0.7454 (2)	0.0270 (6)	
C5	0.17915 (10)	0.1560 (3)	1.0408 (2)	0.0248 (6)	
C6	0.17718 (11)	0.2247 (3)	1.1548 (2)	0.0281 (6)	
H6	0.1462	0.2791	1.1693	0.034*	
C7	0.21958 (11)	0.2150 (3)	1.2473 (3)	0.0308 (6)	
H7	0.2177	0.2624	1.3248	0.037*	
C8	0.26506 (10)	0.1355 (3)	1.2265 (3)	0.0287 (6)	
C9	0.26789 (11)	0.0656 (3)	1.1132 (3)	0.0292 (6)	
H9	0.2990	0.0118	1.0988	0.035*	
C10	0.22495 (11)	0.0750 (3)	1.0215 (3)	0.0286 (6)	
H10	0.2265	0.0260	0.9445	0.034*	
C11	0.30895 (11)	0.1252 (3)	1.3245 (3)	0.0350 (7)	
C12	0.02472 (10)	0.3973 (3)	0.7823 (2)	0.0228 (5)	
C13	0.04203 (10)	0.5483 (3)	0.8172 (3)	0.0274 (6)	
C14	0.0000	0.6490 (4)	0.7500	0.0306 (8)	
C15	0.04599 (12)	0.1178 (3)	0.6201 (3)	0.0331 (6)	
H15A	0.0714	0.0602	0.5775	0.040*	
H15B	0.0358	0.2039	0.5704	0.040*	
H15C	0.0138	0.0603	0.6302	0.040*	

### Atomic displacement parameters (Å^2^)

**Table:**

	*U*^11^	*U*^22^	*U*^33^	*U*^12^	*U*^13^	*U*^23^
S1	0.0312 (4)	0.0264 (4)	0.0297 (4)	0.0058 (3)	−0.0006 (3)	−0.0016 (3)
F1	0.0697 (13)	0.0316 (9)	0.0346 (10)	0.0099 (8)	−0.0174 (9)	−0.0094 (7)
F2	0.0327 (10)	0.0317 (10)	0.122 (2)	−0.0050 (8)	0.0302 (11)	0.0038 (11)
F3	0.0843 (17)	0.0847 (17)	0.0741 (15)	0.0586 (14)	−0.0430 (13)	−0.0557 (13)
N1	0.0378 (15)	0.0602 (18)	0.0466 (16)	−0.0076 (13)	−0.0110 (12)	0.0141 (14)
C1	0.0246 (12)	0.0227 (12)	0.0283 (13)	0.0003 (10)	0.0023 (10)	0.0013 (10)
C2	0.0240 (12)	0.0234 (12)	0.0269 (13)	0.0005 (10)	0.0017 (10)	0.0004 (10)
C3	0.0232 (12)	0.0235 (13)	0.0263 (13)	−0.0004 (10)	0.0012 (10)	0.0023 (10)
C4	0.0277 (13)	0.0263 (13)	0.0268 (13)	0.0018 (10)	0.0012 (10)	0.0016 (11)
C5	0.0233 (12)	0.0221 (12)	0.0289 (13)	−0.0006 (10)	0.0016 (10)	0.0044 (10)
C6	0.0272 (13)	0.0262 (13)	0.0306 (14)	0.0038 (10)	0.0011 (10)	0.0026 (11)
C7	0.0357 (15)	0.0266 (14)	0.0292 (14)	−0.0003 (11)	−0.0012 (11)	0.0032 (11)
C8	0.0260 (13)	0.0267 (13)	0.0324 (14)	−0.0014 (10)	−0.0023 (11)	0.0096 (11)
C9	0.0229 (13)	0.0280 (14)	0.0366 (15)	0.0022 (10)	0.0022 (11)	0.0064 (11)
C10	0.0282 (13)	0.0275 (13)	0.0301 (14)	0.0022 (10)	0.0033 (11)	0.0025 (11)
C11	0.0304 (14)	0.0348 (15)	0.0393 (16)	−0.0022 (12)	0.0007 (13)	0.0072 (13)
C12	0.0249 (12)	0.0219 (12)	0.0216 (12)	−0.0018 (10)	0.0032 (10)	0.0007 (10)
C13	0.0218 (13)	0.0261 (13)	0.0342 (14)	−0.0021 (10)	0.0027 (11)	−0.0006 (11)
C14	0.038 (2)	0.0211 (18)	0.032 (2)	0.000	−0.0003 (16)	0.000
C15	0.0375 (15)	0.0310 (15)	0.0292 (14)	0.0076 (12)	−0.0038 (12)	−0.0051 (12)

### Geometric parameters (Å, °)

**Table:**

S1—C4	1.724 (3)	C6—H6	0.9500
S1—C1	1.732 (3)	C7—C8	1.392 (4)
F1—C13	1.363 (3)	C7—H7	0.9500
F2—C13	1.341 (3)	C8—C9	1.390 (4)
F3—C14	1.331 (3)	C8—C11	1.446 (4)
N1—C11	1.144 (4)	C9—C10	1.384 (4)
C1—C2	1.367 (4)	C9—H9	0.9500
C1—C5	1.465 (3)	C10—H10	0.9500
C2—C3	1.427 (4)	C12—C12^i^	1.354 (5)
C2—H2	0.9500	C12—C13	1.502 (3)
C3—C4	1.380 (4)	C13—C14	1.531 (3)
C3—C12	1.467 (3)	C14—F3^i^	1.331 (3)
C4—C15	1.493 (4)	C14—C13^i^	1.531 (3)
C5—C6	1.389 (4)	C15—H15A	0.9800
C5—C10	1.402 (4)	C15—H15B	0.9800
C6—C7	1.380 (4)	C15—H15C	0.9800
			
C4—S1—C1	93.15 (13)	C8—C9—H9	120.3
C2—C1—C5	127.3 (2)	C9—C10—C5	120.8 (3)
C2—C1—S1	110.0 (2)	C9—C10—H10	119.6
C5—C1—S1	122.54 (19)	C5—C10—H10	119.6
C1—C2—C3	113.8 (2)	N1—C11—C8	177.0 (3)
C1—C2—H2	123.1	C12^i^—C12—C3	131.76 (14)
C3—C2—H2	123.1	C12^i^—C12—C13	110.66 (14)
C4—C3—C2	112.6 (2)	C3—C12—C13	117.6 (2)
C4—C3—C12	125.3 (2)	F2—C13—F1	104.8 (2)
C2—C3—C12	121.7 (2)	F2—C13—C12	113.5 (2)
C3—C4—C15	130.6 (2)	F1—C13—C12	111.3 (2)
C3—C4—S1	110.39 (19)	F2—C13—C14	112.1 (2)
C15—C4—S1	118.9 (2)	F1—C13—C14	108.70 (19)
C6—C5—C10	118.7 (2)	C12—C13—C14	106.5 (2)
C6—C5—C1	119.6 (2)	F3^i^—C14—F3	108.2 (4)
C10—C5—C1	121.6 (2)	F3^i^—C14—C13^i^	111.45 (14)
C7—C6—C5	120.9 (2)	F3—C14—C13^i^	110.49 (15)
C7—C6—H6	119.5	F3^i^—C14—C13	110.49 (15)
C5—C6—H6	119.5	F3—C14—C13	111.45 (14)
C6—C7—C8	119.8 (3)	C13^i^—C14—C13	104.8 (3)
C6—C7—H7	120.1	C4—C15—H15A	109.5
C8—C7—H7	120.1	C4—C15—H15B	109.5
C9—C8—C7	120.3 (2)	H15A—C15—H15B	109.5
C9—C8—C11	120.5 (2)	C4—C15—H15C	109.5
C7—C8—C11	119.2 (3)	H15A—C15—H15C	109.5
C10—C9—C8	119.4 (2)	H15B—C15—H15C	109.5
C10—C9—H9	120.3		
			
C4—S1—C1—C2	0.4 (2)	C8—C9—C10—C5	1.1 (4)
C4—S1—C1—C5	−176.3 (2)	C6—C5—C10—C9	−1.2 (4)
C5—C1—C2—C3	176.4 (2)	C1—C5—C10—C9	175.4 (2)
S1—C1—C2—C3	−0.1 (3)	C4—C3—C12—C12^i^	37.9 (5)
C1—C2—C3—C4	−0.3 (3)	C2—C3—C12—C12^i^	−149.8 (3)
C1—C2—C3—C12	−173.6 (2)	C4—C3—C12—C13	−140.0 (3)
C2—C3—C4—C15	−176.1 (3)	C2—C3—C12—C13	32.3 (3)
C12—C3—C4—C15	−3.2 (5)	C12^i^—C12—C13—F2	−131.7 (3)
C2—C3—C4—S1	0.6 (3)	C3—C12—C13—F2	46.7 (3)
C12—C3—C4—S1	173.5 (2)	C12^i^—C12—C13—F1	110.5 (3)
C1—S1—C4—C3	−0.6 (2)	C3—C12—C13—F1	−71.2 (3)
C1—S1—C4—C15	176.6 (2)	C12^i^—C12—C13—C14	−7.9 (3)
C2—C1—C5—C6	21.9 (4)	C3—C12—C13—C14	170.46 (19)
S1—C1—C5—C6	−162.0 (2)	F2—C13—C14—F3^i^	7.2 (3)
C2—C1—C5—C10	−154.7 (3)	F1—C13—C14—F3^i^	122.5 (2)
S1—C1—C5—C10	21.4 (3)	C12—C13—C14—F3^i^	−117.5 (3)
C10—C5—C6—C7	0.6 (4)	F2—C13—C14—F3	−113.2 (3)
C1—C5—C6—C7	−176.0 (2)	F1—C13—C14—F3	2.2 (3)
C5—C6—C7—C8	0.0 (4)	C12—C13—C14—F3	122.2 (3)
C6—C7—C8—C9	−0.1 (4)	F2—C13—C14—C13^i^	127.3 (3)
C6—C7—C8—C11	−179.4 (3)	F1—C13—C14—C13^i^	−117.3 (2)
C7—C8—C9—C10	−0.4 (4)	C12—C13—C14—C13^i^	2.69 (11)
C11—C8—C9—C10	178.9 (2)		

Symmetry codes: (i) −*x*, *y*, −*z*+3/2.
